# A one-dimensional triaqua­europium(III)–1*H*,3*H*-benzimidazol-3-ium-5,6-dicarboxyl­ate–sulfate polymeric structure

**DOI:** 10.1107/S1600536811034337

**Published:** 2011-08-27

**Authors:** Xia Cai, Jing-Jun Lin, Hao-Zhao Chen, Lai-Chen Chen, Rong-Hua Zeng

**Affiliations:** a School of Chemistry and Environment, South China Normal University, Guangzhou 510006, People’s Republic of China; b Key Laboratory of Technology of Electrochemical Energy Storage and Power Generation in Guangdong Universities, South China Normal University, Guangzhou 510006, People’s Republic of China

## Abstract

In the title coordination polymer, *catena*-poly[[[triaqua­europium(III)]-bis­(μ-1*H*,3*H*-benzimidazol-3-ium-5,6-dicarb­oxyl­ato-κ^3^ *O* ^5^,*O* ^5′^:*O* ^6^)-[triaqua­europium(III)]-di-μ-sulfato-κ^3^ *O*:*O*,*O*′;κ^3^ *O*,*O*′:*O*′] hexahydrate], [Eu_2_(C_9_H_5_N_2_O_4_)_2_(SO_4_)_2_(H_2_O)_6_]·6H_2_O}_*n*_, the 1*H*,3*H*-benzimidazol-3-ium-5,6-dicarb­oxy­l­ate ligand is protonated at the imidazole group (H_2_bdc). The Eu^III^ ion is coordinated by nine O atoms from two H_2_bdc ligands, two sulfate anions and three water mol­ecules, displaying a bicapped trigonal prismatic geometry. The carboxyl­ate groups of the H_2_bdc ligands and the sulfate anions link the Eu^III^ ions, forming a chain along [010]. These chains are further connected by N—H⋯O and O—H⋯O hydrogen bonds and π–π inter­actions between the imidazole and benzene rings [centroid–centroid distances = 3.997 (4), 3.829 (4) and 3.573 (4) Å] into a three-dimensional supra­molecular network.

## Related literature

For background to 1*H*-benzimidazole-5,6-dicarboxyl­ate complexes, see: Wang *et al.* (2010 ▶); Wei *et al.* (2008 ▶); Xie *et al.* (2010 ▶); Yao *et al.* (2008 ▶).
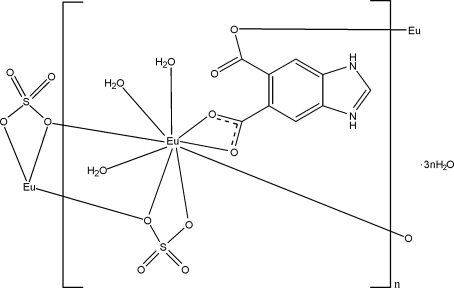


## Experimental

### 

#### Crystal data

[Eu_2_(C_9_H_5_N_2_O_4_)_2_(SO_4_)_2_(H_2_O)_6_]·6H_2_O
*M* *_r_* = 1122.58
Triclinic, 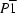

*a* = 7.1261 (16) Å
*b* = 9.581 (2) Å
*c* = 12.424 (3) Å
α = 100.496 (3)°
β = 98.060 (3)°
γ = 94.979 (3)°
*V* = 820.3 (3) Å^3^
*Z* = 1
Mo *K*α radiation
μ = 4.03 mm^−1^
*T* = 298 K
0.30 × 0.26 × 0.20 mm

#### Data collection

Bruker APEXII CCD diffractometer
Absorption correction: multi-scan (*SADABS*; Bruker, 2001 ▶) *T*_min_ = 0.310, *T*_max_ = 0.446
4076 measured reflections
2841 independent reflections
2669 reflections with *I* > 2σ(*I*)
*R*_int_ = 0.024

#### Refinement

*R*[*F* ^2^ > 2σ(*F* ^2^)] = 0.036
*wR*(*F* ^2^) = 0.097
*S* = 1.02
2841 reflections
248 parameters
H atoms treated by a mixture of independent and constrained refinement
Δρ_max_ = 1.82 e Å^−3^
Δρ_min_ = −2.51 e Å^−3^

### 

Data collection: *APEX2* (Bruker, 2007 ▶); cell refinement: *SAINT* (Bruker, 2007 ▶); data reduction: *SAINT*; program(s) used to solve structure: *SHELXS97* (Sheldrick, 2008 ▶); program(s) used to refine structure: *SHELXL97* (Sheldrick, 2008 ▶); molecular graphics: *ORTEPIII* (Burnett & Johnson, 1996 ▶) and *PLATON* (Spek, 2009 ▶); software used to prepare material for publication: *SHELXL97*.

## Supplementary Material

Crystal structure: contains datablock(s) global. DOI: 10.1107/S1600536811034337/hy2456sup1.cif

Additional supplementary materials:  crystallographic information; 3D view; checkCIF report

## Figures and Tables

**Table 1:** Hydrogen-bond geometry (Å, °)

*D*—H⋯*A*	*D*—H	H⋯*A*	*D*⋯*A*	*D*—H⋯*A*
O1*W*—H1*W*⋯O4^i^	0.84	1.90	2.717 (6)	165
O1*W*—H2*W*⋯O7^ii^	0.84	2.24	3.049 (6)	163
O2*W*—H3*W*⋯O5^ii^	0.84	1.96	2.775 (6)	162
O2*W*—H4*W*⋯O3^iii^	0.85	1.85	2.659 (6)	159
O3*W*—H5*W*⋯O4*W*^iii^	0.84	2.07	2.810 (6)	146
O3*W*—H6*W*⋯O2^iv^	0.85	2.10	2.864 (6)	149
O4*W*—H7*W*⋯O5^ii^	0.86	2.34	3.045 (6)	139
O4*W*—H8*W*⋯O1	0.84	2.04	2.869 (6)	168
O5*W*—H9*W*⋯O6*W*	0.84	2.03	2.864 (8)	171
O5*W*—H10*W*⋯O6^v^	0.84	2.01	2.790 (7)	154
O6*W*—H11*W*⋯O6^vi^	0.84	2.37	3.165 (8)	158
O6*W*—H12*W*⋯O5^ii^	0.85	2.20	2.895 (7)	139
O6*W*—H12*W*⋯O1*W*	0.85	2.46	3.060 (7)	129
N1—H1*A*⋯O5*W*^vii^	0.86 (8)	1.96 (8)	2.752 (8)	153 (7)
N1—H1*A*⋯O4*W*^vii^	0.86 (8)	2.48 (8)	2.989 (7)	119 (6)
N2—H2⋯O6*W*^viii^	0.86	1.91	2.734 (7)	161

Symmetry codes: (i) 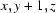; (ii) 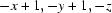; (iii) 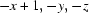; (iv) 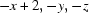; (v) 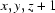; (vi) 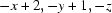; (vii) 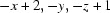; (viii) 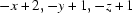.

**Fig. 1.**
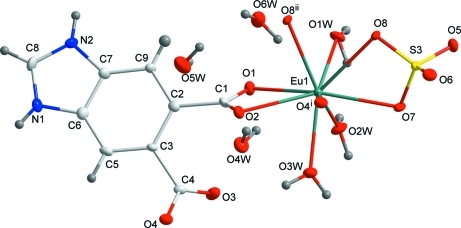
The asymmetric unit of the title compound. Displacement ellipsoids are drawn at the 30% probability level. [Symmetry codes: (i) 2-x, -y, -z; (ii) 2-x, 1-y, -z.]

**Fig. 2.**
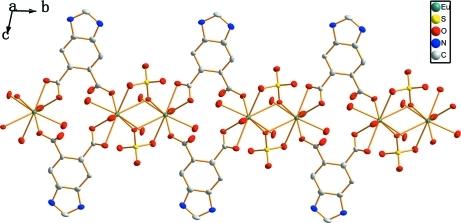
A view of the chain structure along [0 1 0].

## supplementary crystallographic information

### Comment

In recent years, research on coordination polymers has made considerable progress in the fields of supramolecular chemistry and crystal engineering, because of their intriguing structural motifs and functional properties, such as molecular adsorption, magnetism and luminescence. Ligands play a key role in the construction of coordination polymers with fascinating topology, intriguing architectures and useful physical-chemical properties. Benzimidazole-5,6-dicarboxylic acid (H_3_bdc) is a potential bifunctional ligand with carboxylate and N-donor functional groups and has been used to prepare such metal-organic complexes in possession of multidimensional networks and interesting properties (Wang *et al.*, 2010; Wei *et al.*, 2008; Xie *et al.*, 2010; Yao *et al.*, 2008). Recently, we obtained the title coordination polymer, which was synthesized by the hydrothermal reaction of Eu_2_O_3_ with H_3_bdc in an aqueous solution.

The title compound has a polymeric chain architecture. As shown in Fig. 1,  the Eu^III^ ion is in a bicapped trigonal-prismatic geometry, defined by nine O atoms from two 1*H*,3*H*-benzimidazol-3-ium-5,6-dicarboxylate ligands (H_2_bdc), which are protonated at the imidazole groups, two sulfate anions and three water molecules. The H_2_bdc ligands and sulfate anions link the Eu^III^ ions into a chain along [0 1 0] (Fig. 2). The adjacent Eu···Eu separations are 4.272 (4) and 6.663 (5) Å. The Eu—O bond lengths range from 2.376 (4) to 2.610 (4) Å and O—Eu—O bond angles vary from 52.01 (1) to 143.68 (1) °. The chains are further connected by N—H···O and O—H···O hydrogen bonds (Table 1) and π–π interactions between the imidazole and benzene rings [centroid–centroid distances = 3.997 (4), 3.829 (4) and 3.573 (4) Å] into a three-dimensional supramolecular network.

### Experimental

A mixture of Eu_2_O_3_ (0.352 g, 1 mmol), H_3_bdc (0.206 g, 1 mmol), water (10 ml) in the presence of H_2_SO_4_ (0.038 g, 0.385 mmol) was stirred vigorously for 30 min and then sealed in a 20 ml Teflon-lined stainless-steel autoclave. The autoclave was heated and maintained at 443 K for 3 days, and then cooled to room temperature at 5 K h^-1^. Colorless block crystals of the title compound were obtained.

### Refinement

Water H atoms were tentatively located in difference Fourier maps and were refined with distance restraints of O—H = 0.84 and H···H = 1.35 Å, and with *U*_iso_(H) = 1.5*U*_eq_(O). H atoms bound to C and N atoms were positioned geometrically and refined as riding atoms, with C—H = 0.93 and N—H = 0.86 Å and with *U*_iso_(H) = 1.2*U*_eq_(C, N). H1A atom attached to N1 was refined with *U*_iso_(H) = 0.035 Å^2^. The highest residual electron density was found at 1.00 Å from Eu1 atom and the deepest hole at 0.97 Å from Eu1 atom.

### Crystal data

**Table:**

[Eu_2_(C_9_H_5_N_2_O_4_)_2_(SO_4_)_2_(H_2_O)_6_]·6H_2_O	*Z* = 1
*M**_r_* = 1122.58	*F*(000) = 552
Triclinic, *P*1	*D*_x_ = 2.273 Mg m^−^^3^
Hall symbol: -P 1	Mo *K*α radiation, λ = 0.71073 Å
*a* = 7.1261 (16) Å	Cell parameters from 3240 reflections
*b* = 9.581 (2) Å	θ = 2.5–25.2°
*c* = 12.424 (3) Å	µ = 4.03 mm^−^^1^
α = 100.496 (3)°	*T* = 298 K
β = 98.060 (3)°	Block, colorless
γ = 94.979 (3)°	0.30 × 0.26 × 0.20 mm
*V* = 820.3 (3) Å^3^	

### Data collection

**Table:**

Bruker APEXII CCD diffractometer	2841 independent reflections
Radiation source: fine-focus sealed tube	2669 reflections with *I* > 2σ(*I*)
graphite	*R*_int_ = 0.024
φ and ω scans	θ_max_ = 25.2°, θ_min_ = 1.7°
Absorption correction: multi-scan (*SADABS*; Bruker, 2001)	*h* = −8→8
*T*_min_ = 0.310, *T*_max_ = 0.446	*k* = −10→11
4076 measured reflections	*l* = −14→14

### Refinement

**Table:**

Refinement on *F*^2^	Primary atom site location: structure-invariant direct methods
Least-squares matrix: full	Secondary atom site location: difference Fourier map
*R*[*F*^2^ > 2σ(*F*^2^)] = 0.036	Hydrogen site location: inferred from neighbouring sites
*wR*(*F*^2^) = 0.097	H atoms treated by a mixture of independent and constrained refinement
*S* = 1.02	*w* = 1/[σ^2^(*F*_o_^2^) + (0.0656*P*)^2^ + 2.2735*P*] where *P* = (*F*_o_^2^ + 2*F*_c_^2^)/3
2841 reflections	(Δ/σ)_max_ < 0.001
248 parameters	Δρ_max_ = 1.82 e Å^−^^3^
0 restraints	Δρ_min_ = −2.51 e Å^−^^3^

### Fractional atomic coordinates and isotropic or equivalent isotropic displacement parameters (Å^2^)

**Table:**

	*x*	*y*	*z*	*U*_iso_*/*U*_eq_	
Eu1	0.82011 (3)	0.30949 (3)	−0.00038 (2)	0.01547 (13)	
S3	0.7557 (2)	0.48078 (15)	−0.19659 (11)	0.0178 (3)	
O1	0.8381 (6)	0.2686 (5)	0.1916 (3)	0.0245 (9)	
O2	1.0266 (6)	0.1509 (4)	0.0920 (3)	0.0235 (9)	
O7	0.6411 (6)	0.3533 (5)	−0.1756 (3)	0.0255 (9)	
O8	0.9029 (5)	0.5245 (4)	−0.0939 (3)	0.0197 (8)	
O5	0.6331 (6)	0.5948 (5)	−0.2072 (3)	0.0274 (9)	
O6	0.8483 (7)	0.4467 (5)	−0.2935 (3)	0.0286 (10)	
N1	1.3014 (7)	−0.0260 (6)	0.5455 (4)	0.0249 (11)	
N2	1.2981 (7)	0.2039 (6)	0.5675 (4)	0.0251 (11)	
H2	1.3174	0.2932	0.5979	0.030*	
C1	0.9614 (8)	0.1832 (6)	0.1807 (5)	0.0190 (12)	
C2	1.0383 (8)	0.1201 (6)	0.2791 (5)	0.0194 (12)	
C3	1.0338 (8)	−0.0312 (6)	0.2662 (4)	0.0164 (11)	
C5	1.1219 (8)	−0.0927 (6)	0.3485 (5)	0.0198 (12)	
H5	1.1239	−0.1911	0.3393	0.024*	
C6	1.2079 (8)	−0.0005 (6)	0.4462 (5)	0.0218 (12)	
C7	1.2050 (8)	0.1468 (6)	0.4603 (5)	0.0213 (12)	
C8	1.3518 (9)	0.0977 (7)	0.6146 (5)	0.0274 (14)	
H8	1.4162	0.1086	0.6866	0.033*	
C9	1.1232 (8)	0.2109 (6)	0.3771 (4)	0.0189 (11)	
H9	1.1250	0.3095	0.3864	0.023*	
O4W	0.5121 (7)	0.1310 (5)	0.2584 (4)	0.0352 (11)	
H8W	0.6008	0.1653	0.2294	0.053*	
H7W	0.4457	0.2009	0.2736	0.053*	
O6W	0.7298 (9)	0.5166 (6)	0.3452 (4)	0.0439 (13)	
H11W	0.8442	0.5054	0.3395	0.066*	
H12W	0.6655	0.4779	0.2826	0.09 (4)*	
O5W	0.6913 (11)	0.3173 (6)	0.4890 (5)	0.0644 (19)	
H9W	0.6934	0.3808	0.4506	0.097*	
H10W	0.7710	0.3559	0.5453	0.097*	
O3W	0.7114 (6)	0.0596 (4)	−0.0781 (4)	0.0283 (10)	
H5W	0.6097	0.0179	−0.1180	0.042*	
H6W	0.7713	−0.0084	−0.0605	0.042*	
O1W	0.7080 (6)	0.5224 (4)	0.0984 (4)	0.0266 (9)	
H2W	0.6011	0.5504	0.1051	0.040*	
H1W	0.7804	0.5996	0.1181	0.040*	
O2W	0.4946 (6)	0.2615 (5)	0.0214 (4)	0.0308 (10)	
H4W	0.4110	0.1952	−0.0161	0.046*	
H3W	0.4346	0.3063	0.0678	0.046*	
C4	0.9079 (8)	−0.1258 (6)	0.1673 (4)	0.0179 (11)	
O4	0.9655 (6)	−0.2426 (4)	0.1255 (3)	0.0226 (9)	
O3	0.7505 (6)	−0.0878 (5)	0.1382 (4)	0.0290 (10)	
H1A	1.323 (11)	−0.109 (9)	0.557 (6)	0.035*	

### Atomic displacement parameters (Å^2^)

**Table:**

	*U*^11^	*U*^22^	*U*^33^	*U*^12^	*U*^13^	*U*^23^
Eu1	0.01833 (18)	0.01343 (19)	0.01383 (18)	0.00052 (11)	0.00124 (11)	0.00220 (12)
S3	0.0222 (7)	0.0163 (7)	0.0138 (6)	−0.0006 (5)	0.0006 (5)	0.0030 (5)
O1	0.031 (2)	0.026 (2)	0.018 (2)	0.0101 (18)	0.0039 (17)	0.0053 (17)
O2	0.032 (2)	0.022 (2)	0.018 (2)	0.0070 (18)	0.0074 (17)	0.0046 (17)
O7	0.027 (2)	0.021 (2)	0.024 (2)	−0.0095 (17)	−0.0053 (17)	0.0065 (17)
O8	0.021 (2)	0.020 (2)	0.0140 (19)	−0.0041 (16)	−0.0042 (15)	0.0017 (16)
O5	0.034 (2)	0.023 (2)	0.024 (2)	0.0087 (18)	−0.0010 (18)	0.0051 (18)
O6	0.042 (3)	0.025 (2)	0.018 (2)	0.0020 (19)	0.0083 (18)	0.0016 (17)
N1	0.029 (3)	0.027 (3)	0.021 (3)	0.005 (2)	0.004 (2)	0.011 (2)
N2	0.033 (3)	0.022 (3)	0.016 (2)	−0.003 (2)	0.002 (2)	−0.001 (2)
C1	0.026 (3)	0.013 (3)	0.018 (3)	0.001 (2)	0.001 (2)	0.004 (2)
C2	0.018 (3)	0.020 (3)	0.020 (3)	0.000 (2)	0.004 (2)	0.004 (2)
C3	0.019 (3)	0.013 (3)	0.017 (3)	−0.001 (2)	0.003 (2)	0.002 (2)
C5	0.024 (3)	0.013 (3)	0.024 (3)	0.002 (2)	0.007 (2)	0.002 (2)
C6	0.024 (3)	0.022 (3)	0.020 (3)	0.000 (2)	0.005 (2)	0.007 (2)
C7	0.025 (3)	0.022 (3)	0.015 (3)	−0.002 (2)	0.005 (2)	0.002 (2)
C8	0.028 (3)	0.039 (4)	0.013 (3)	0.001 (3)	−0.002 (2)	0.007 (3)
C9	0.025 (3)	0.015 (3)	0.016 (3)	−0.001 (2)	0.005 (2)	0.002 (2)
O4W	0.035 (2)	0.037 (3)	0.039 (3)	0.003 (2)	0.012 (2)	0.014 (2)
O6W	0.067 (4)	0.036 (3)	0.024 (3)	0.016 (3)	−0.003 (2)	−0.002 (2)
O5W	0.123 (6)	0.031 (3)	0.033 (3)	0.003 (3)	−0.006 (3)	0.007 (2)
O3W	0.030 (2)	0.019 (2)	0.032 (2)	−0.0014 (18)	−0.0022 (18)	0.0008 (18)
O1W	0.024 (2)	0.014 (2)	0.043 (3)	0.0046 (16)	0.0111 (19)	0.0037 (18)
O2W	0.023 (2)	0.035 (3)	0.028 (2)	−0.0041 (18)	0.0029 (18)	−0.0072 (19)
C4	0.024 (3)	0.015 (3)	0.015 (3)	−0.003 (2)	0.007 (2)	0.002 (2)
O4	0.030 (2)	0.019 (2)	0.019 (2)	0.0003 (17)	0.0081 (17)	−0.0012 (16)
O3	0.024 (2)	0.025 (2)	0.032 (2)	0.0055 (18)	−0.0039 (18)	−0.0061 (19)

### Geometric parameters (Å, °)

**Table:**

Eu1—O4^i^	2.374 (4)	C3—C5	1.375 (8)
Eu1—O2W	2.387 (4)	C3—C4	1.510 (8)
Eu1—O3W	2.427 (4)	C5—C6	1.392 (8)
Eu1—O8^ii^	2.434 (4)	C5—H5	0.9300
Eu1—O1W	2.439 (4)	C6—C7	1.392 (9)
Eu1—O1	2.474 (4)	C7—C9	1.381 (8)
Eu1—O2	2.518 (4)	C8—H8	0.9300
Eu1—O8	2.607 (4)	C9—H9	0.9300
S3—O6	1.451 (4)	O4W—H8W	0.8415
S3—O5	1.470 (4)	O4W—H7W	0.8612
S3—O7	1.493 (4)	O6W—H11W	0.8423
S3—O8	1.502 (4)	O6W—H12W	0.8471
O1—C1	1.256 (7)	O5W—H9W	0.8390
O2—C1	1.252 (7)	O5W—H10W	0.8403
N1—C8	1.319 (8)	O3W—H5W	0.8408
N1—C6	1.390 (8)	O3W—H6W	0.8534
N1—H1A	0.86 (8)	O1W—H2W	0.8393
N2—C8	1.322 (8)	O1W—H1W	0.8396
N2—C7	1.392 (7)	O2W—H4W	0.8507
N2—H2	0.8600	O2W—H3W	0.8434
C1—C2	1.517 (8)	C4—O3	1.235 (7)
C2—C9	1.387 (8)	C4—O4	1.272 (7)
C2—C3	1.426 (8)		
			
O4^i^—Eu1—O2W	140.91 (14)	C8—N1—C6	108.3 (5)
O4^i^—Eu1—O3W	76.22 (15)	C8—N1—H1A	127 (5)
O2W—Eu1—O3W	71.09 (15)	C6—N1—H1A	124 (5)
O4^i^—Eu1—O8^ii^	81.63 (13)	C8—N2—C7	108.3 (5)
O2W—Eu1—O8^ii^	137.26 (14)	C8—N2—H2	125.8
O3W—Eu1—O8^ii^	143.77 (14)	C7—N2—H2	125.8
O4^i^—Eu1—O1W	140.46 (14)	O2—C1—O1	122.2 (5)
O2W—Eu1—O1W	69.33 (15)	O2—C1—C2	118.8 (5)
O3W—Eu1—O1W	140.36 (14)	O1—C1—C2	119.0 (5)
O8^ii^—Eu1—O1W	71.68 (14)	C9—C2—C3	121.4 (5)
O4^i^—Eu1—O1	126.79 (14)	C9—C2—C1	119.3 (5)
O2W—Eu1—O1	75.86 (15)	C3—C2—C1	119.1 (5)
O3W—Eu1—O1	92.05 (14)	C5—C3—C2	121.3 (5)
O8^ii^—Eu1—O1	78.65 (13)	C5—C3—C4	119.1 (5)
O1W—Eu1—O1	76.39 (14)	C2—C3—C4	119.1 (5)
O4^i^—Eu1—O2	75.47 (14)	C3—C5—C6	116.7 (5)
O2W—Eu1—O2	111.08 (15)	C3—C5—H5	121.6
O3W—Eu1—O2	69.40 (14)	C6—C5—H5	121.6
O8^ii^—Eu1—O2	77.54 (13)	N1—C6—C5	131.7 (6)
O1W—Eu1—O2	124.10 (14)	N1—C6—C7	106.4 (5)
O1—Eu1—O2	52.20 (13)	C5—C6—C7	121.8 (5)
O4^i^—Eu1—O8	71.41 (13)	C9—C7—N2	131.6 (6)
O2W—Eu1—O8	116.64 (14)	C9—C7—C6	122.3 (5)
O3W—Eu1—O8	131.57 (13)	N2—C7—C6	106.2 (5)
O8^ii^—Eu1—O8	64.15 (14)	N1—C8—N2	110.7 (5)
O1W—Eu1—O8	70.87 (13)	N1—C8—H8	124.6
O1—Eu1—O8	136.26 (13)	N2—C8—H8	124.6
O2—Eu1—O8	131.95 (13)	C7—C9—C2	116.4 (5)
O4^i^—Eu1—Eu1^ii^	73.90 (10)	C7—C9—H9	121.8
O2W—Eu1—Eu1^ii^	133.79 (11)	C2—C9—H9	121.8
O3W—Eu1—Eu1^ii^	149.81 (11)	H8W—O4W—H7W	104.3
O8^ii^—Eu1—Eu1^ii^	33.30 (9)	H11W—O6W—H12W	105.6
O1W—Eu1—Eu1^ii^	67.72 (10)	H9W—O5W—H10W	101.4
O1—Eu1—Eu1^ii^	109.19 (10)	Eu1—O3W—H5W	132.6
O2—Eu1—Eu1^ii^	106.55 (10)	Eu1—O3W—H6W	122.9
O8—Eu1—Eu1^ii^	30.84 (8)	H5W—O3W—H6W	104.1
O6—S3—O5	111.0 (3)	Eu1—O1W—H2W	135.5
O6—S3—O7	112.3 (3)	Eu1—O1W—H1W	120.5
O5—S3—O7	109.6 (3)	H2W—O1W—H1W	101.5
O6—S3—O8	110.1 (3)	Eu1—O2W—H4W	128.4
O5—S3—O8	110.2 (2)	Eu1—O2W—H3W	128.4
O7—S3—O8	103.5 (2)	H4W—O2W—H3W	103.2
C1—O1—Eu1	93.8 (3)	O3—C4—O4	125.0 (5)
C1—O2—Eu1	91.8 (3)	O3—C4—C3	116.9 (5)
S3—O8—Eu1^ii^	146.0 (2)	O4—C4—C3	117.9 (5)
S3—O8—Eu1	97.84 (18)	C4—O4—Eu1^i^	135.3 (4)
Eu1^ii^—O8—Eu1	115.85 (14)		

Symmetry codes: (i) −*x*+2, −*y*, −*z*; (ii) −*x*+2, −*y*+1, −*z*.

### Hydrogen-bond geometry (Å, °)

**Table:**

*D*—H···*A*	*D*—H	H···*A*	*D*···*A*	*D*—H···*A*
O1W—H1W···O4^iii^	0.84	1.90	2.717 (6)	165
O1W—H2W···O7^iv^	0.84	2.24	3.049 (6)	163
O2W—H3W···O5^iv^	0.84	1.96	2.775 (6)	162
O2W—H4W···O3^v^	0.85	1.85	2.659 (6)	159
O3W—H5W···O4W^v^	0.84	2.07	2.810 (6)	146
O3W—H6W···O2^i^	0.85	2.10	2.864 (6)	149
O4W—H7W···O5^iv^	0.86	2.34	3.045 (6)	139
O4W—H8W···O1	0.84	2.04	2.869 (6)	168
O5W—H9W···O6W	0.84	2.03	2.864 (8)	171
O5W—H10W···O6^vi^	0.84	2.01	2.790 (7)	154
O6W—H11W···O6^ii^	0.84	2.37	3.165 (8)	158
O6W—H12W···O5^iv^	0.85	2.20	2.895 (7)	139
O6W—H12W···O1W	0.85	2.46	3.060 (7)	129
N1—H1A···O5W^vii^	0.86 (8)	1.96 (8)	2.752 (8)	153 (7)
N1—H1A···O4W^vii^	0.86 (8)	2.48 (8)	2.989 (7)	119 (6)
N2—H2···O6W^viii^	0.86	1.91	2.734 (7)	161

Symmetry codes: (iii) *x*, *y*+1, *z*; (iv) −*x*+1, −*y*+1, −*z*; (v) −*x*+1, −*y*, −*z*; (i) −*x*+2, −*y*, −*z*; (vi) *x*, *y*, *z*+1; (ii) −*x*+2, −*y*+1, −*z*; (vii) −*x*+2, −*y*, −*z*+1; (viii) −*x*+2, −*y*+1, −*z*+1.
